# Bestatin attenuates breast cancer stemness by targeting puromycin-sensitive aminopeptidase

**DOI:** 10.1007/s12672-024-01063-4

**Published:** 2024-05-30

**Authors:** Yan Ma, Xintong Yang, Pengge Pan, Jinyi Yang, Xiaojuan Wu, Danhan Wang, Hui Gao

**Affiliations:** 1 Key Laboratory of Fertility Preservation and Maintenance of Ministry of Education, Ningxia Medical University, Yinchuan, 750004 People’s Republic of China; 2 Shengzhou Food and Drug Testing Center, Shaoxing, 312400 China; 3 The 2nd Afflicated Hospital and Yuying Children’s Hospital, Wenzhou Medical University, Wenzhou, 325035 People’s Republic of China

**Keywords:** Breast cancer, Bestatin, Breast cancer stem cells, Puromycin-sensitive aminopeptidase, Apoptosis, Proliferation

## Abstract

Breast cancer is a prevalent malignant tumor among women with an increasing incidence rate annually. Breast cancer stem cells (BCSCs) are integral in impeding tumor advancement and addressing drug resistance. Bestatin serves as an adjuvant chemotherapy, triggering apoptosis in cancer cells. In this study, the effects of bestatin on sorted BCSCs from breast cancer cell lines have been studied. Our results indicated that bestatin inhibits the migration and proliferation of breast cancer cells by reducing the stemness of BCSCs both in vitro and in vivo. Puromycin-sensitive aminopeptidase is implicated in the process through the regulation of cell cycle, resulting in heightened cell apoptosis and diminished cell proliferation of BCSCs. Our study suggest that targeting cancer stem cell may offer a promising approach in breast cancer treatment, presenting noval therapeutic strategies for patients with breast cancer.

## Introduction

Breast cancer becomes the leading cancer incidence and mortality for women since 2020 with a total of 2.26 million new cases and 685 thousand associated deaths yearly, and the 5-year survival rate of metastatic breast cancer is no more than 30% even with adjuvant chemotherapy [1, 2]. Although more effective diagnostic and treatment strategies have improved the prognosis of patients in the past few decades, a considerable number of patients are still difficult to treat with current adjuvant chemotherapy strategies [3–5]. It has been reported that there is an enrichment of cancer stem cells (or named with tumor initiating stem cells) in chemotherapy and/or radiotherapy resistant breast cancer patients [6]. Due to its intrinsic stem cell characteristics of self-renewal and pluripotent abilities, cancer stem cells play an important role in inhibiting tumor progression and treating drug resistance, while traditional chemotherapies often lead to treatment failure due to its inability to eradicate cancer stem cells [7]. Therefore, cancer stem cells might be a new targeted strategy to improve clinical outcomes in breast cancer patients.

As an analog of puromycin, bestatin is an antibiotic of microbial origin which can be used for immuno-modification and anti-tumor therapy [8, 9]. Bestatin can work as an adjuvant modality combined with chemotherapy as an immunomodulatory therapy. Bestatin is usually used as an immune modulator to exert therapeutic effects in combination with chemotherapy and/or radiotherapy, which has been widely used for cancer treatment of respiratory, digestive and immune system [10–12]. In recent years, bestatin was found to be able to act as an anti-tumor drug in the treatment of some types of cancer directly [13, 14]. However, the efficacy of ubenimex in the treatment of breast cancer has not yet been reported.

Bestatin is an aminopeptidase inhibitor and it can inhibit puromycin-sensitive aminopeptidase (PSA, encoded by NPEPPS gene) [15, 16]. PSA is a zinc metallopeptidase with subcellular distribution in the cytosol and nucleus, which hydrolyzes the substrate from N-terminal of amino acids [17]. PSA is proven to be a regulator of Alzheimer’s disease [18]; it was also found to be a pivotal regulator of hepatic lipid metabolism [19], and the capability to promote myoblast proliferation and differentiation was exerted [20]. To the best of our knowledge, few studies have reported the role of PSA in breast cancer yet. Thus, we investigated the relationship between PSA and the stemness of breast cancer cells.

## Materials and methods

### Chemicals

Bestatin (CAS# 58970-76-6, purity: 99.96%) was purchased from MedChemExpress (Shanghai, China) and the stock solutions of 10 mM were prepared by dissolving it in 100% dimethyl sulfoxide (DMSO) and stored at − 20 °C prior to use.

### Cell culture

Mammary breast cancer cell lines MCF-7 and SKRB3 were obtained from the Cell Resource Center, Peking Union Medical College (China). MCF-7 were cultured in DMEM (Gibco; Thermo Fisher Scientific, Inc.) supplemented with 10% fetal bovine serum (Gibco; Thermo Fisher Scientific, Inc.) and SKRB3 were cultured in McCoy's 5A (Gibco; Thermo Fisher Scientific, Inc.) supplemented with 10% fetal bovine serum (Gibco; Thermo Fisher Scientific, Inc.) at 37 °C in a humidified 5% CO_2_ atmosphere.

### Wound healing assay

Cells were cultured until they were 70% confluent in 6‑well plate, then a wound was drawn by a sterile 200 µl pipette tip. Subsequently, cells were cultured in fresh serum-free medium with/without 0.25 mM bestatin for 24 h. Bright-field images of the wounded area were taken after 0 and 24 h at the same microscopic cross point using a Nikon microscope. Wound width was measured was quantified by ImageJ (National Institutes of Health).

### Colony formation assay

Cells were seeded in 6-well plates at 10^3^ cells/well with three repetitions., and a final concentration of 0.25 mM bestatin was added into the medium for treatment group on the second day. Fresh medium was changed every 5 days, and after 2 weeks plates were fixed with methanol and stained with 0.1% crystal violet solution for further analysis under the Nikon microscope.

### Flow cytometry analysis of breast cancer stem cells

After dissociated with 0.05% trypsin/EDTA, harvest cells were resuspended in 200 μL HBSS with 2% FBS. Subsequently, cells were incubated with ALDEFLUOR^™^ (STEMCELL Technologies, cat#01700) at the recommended concentration for 45 min in the dark on the ice, and then resuspended for flow cytometry analysis (BD Aria III, BD Biosciences, USA).

### Sphere formation assay

Sorted breast cancer stem cells were plated in the ultra-low attachment 96-well plate with the basic culture medium supplied with 2% B27 (Invitrogen), 1% penicillin/streptomycin, 10 ng/mL basic fibroblast growth factor, 10 ng/mL EGF, 10 μg/mL insulin, 10 μg/mL heparin and 1 μg/mL hydrocortisone. Spheres were collected after cultured with/without bestatin for 5 days.

### 3D culture of breast cancer stem cells

Sorted breast cancer stem cells were resuspended in Pre-chilled Matrigel (BD Biosciences) in the ultra-low attachment 6 -well plate. After incubated for 15 min in 37 °C incubator, basic culture medium supplied with 10% FBS, 2% B27 (Invitrogen), 1% penicillin/streptomycin, 10 ng/mL basic fibroblast growth factor, 10 ng/mL EGF, 10 μg/mL insulin, 10 μg/mL heparin and 1 μg/mL hydrocortisone were added for 3D formation. The number of 3Ds with/without bestatin treatment was counted after 6 days.

### Immunofluorescence staining of spheres

Spheres collected from ultra-low attachment 96-well plate were washed with HBSS and fixed with 4% paraformaldehyde for 120 min. Then the fixed spheres were dehydrated in ethanol, and embedded in paraffin. Paraffin-embedded sections on glass slides were deparaffinized, rehydrated and microwaved for antigen retrieval. After blocking nonspecific binding, sections were incubated with primary antibodies overnight at 4 °C, followed by incubation with fluorescence-conjugated secondary antibody for 120 min. Sections were stained with DAPI for 10 min and viewed with a confocal microscope.

### Prognosis analysis

The KM plotter database (http://kmplot.com/analysis/) was used to assess the effect of NPEPPS on survival in breast cancer patients on gene and protein levels [21]. HPA database (Human Protein Atlas proteinatlas.org) was also used to obtain the expression profiles at protein levels, as well as IHC images for a wide variety of cancer tissues [22]. Besides, expression levels of PSA gene in breast cancer and the subtypes were verified by BEST database (https://rookieutopia.com/) [23].

### Xenograft mouse model

50 of the spheres formed by MCF-7 BCSCs were collected and injected into cleared fat pad (mammary glands of 3-week-old nude mice cleared of endogenous epithelium). 6 weeks later, mice were injected with 50 mg/kg bestatin or PBS every 2 days for 10 days. The tumor volume was monitored every day by measuring the longest diameter (L) and the shortest diameter (W) with a caliper, and tumor volume was calculated using the formula [24]: Volume = L × W^2^ × 0.5. The mice were euthanized by isoflurane 30 days after injection, tumors and lungs were collected for further pathological analysis. Based on national regulations and guidelines, the 3-week-old female nude mice were purchased from the Experimental Animal Center of Ningxia Medical University (Yinchuan, China). The study protocol was conducted in accordance with the principles and procedure granted by the Ethics Committee of Ningxia Medical University (No. 2022-G220, Approved Date: 10 March, 2022) and all methods were followed the Care and Use of Laboratory Animals Principles of Ningxia Medical University. The maximal tumor size of mice permitted by the Ethics Committee of Ningxia Medical University is no more than 20 mm in any dimension, and the tumor size in this study was not exceeded than the maximal tumor size approved.

### Quantification of lung metastasis

After infusing by 4% paraformaldehyde, lungs of xenograft mice were collected for histological analysis. The tissues were embedded in paraffin, and the sections were stained with hematoxylin and eosin. All images of the sections were collected by Nikon microscope and the number of metastatic lesions in the tissue sections was counted.

### Immunohistochemistry staining

Fixed tumors were embedded in paraffin wax and sections of 4 μm were plated on glass slides. After antigen retrieval and blocking of non-specific binding, sections were incubated with incubated with primary antibodies overnight at 4 °C, and then washed twice and incubated with secondary antibodies for 1 h at room temperature. Sections were incubated with streptavidin-horseradish peroxidases for 25 min and stained with diaminobenzidine for 10 min before dehydration and mounting. Integral optical density (IOD) of the sections was measured by ImageJ software.

### Statistical analysis

Statistical analysis was performed by GraphPad prism version 8.0.2. Results are presented as mean ± standard deviation (SD). The difference between two groups was analyzed by the Student’s t test, and the difference among multiple groups was analyzed by ANOVA. A *p*-value less than 0.05 was considered to be statistically significant.

## Results

### Bestatin inhibits the migration, proliferation and cell cycles of breast cancer cells

**Fig. 1:**
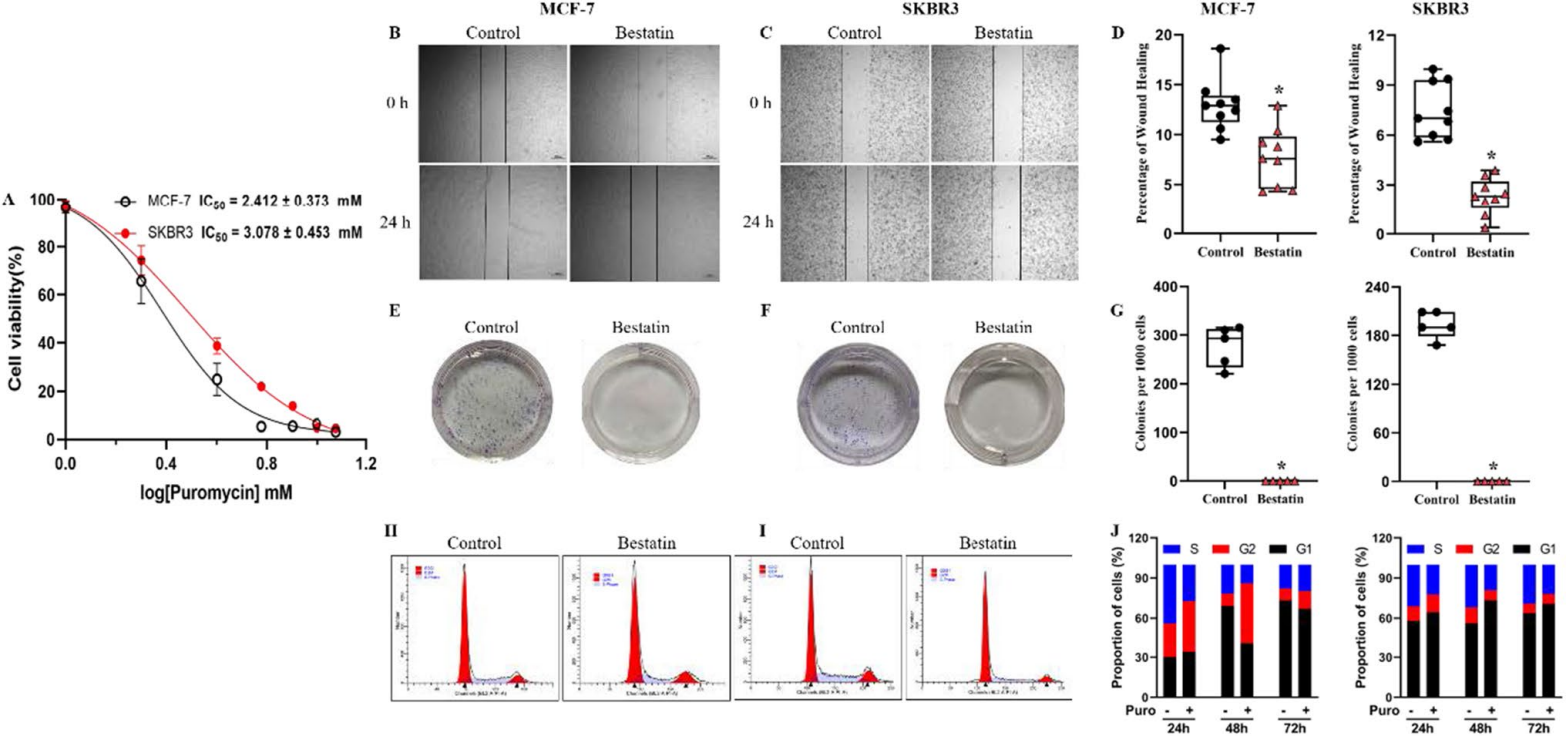
Effects of bestatin on migration, proliferation and cell cycles for MCF-7 and SKBR3 breast cancer cell lines. **A** A dose–response curve for bestatin in MCF-7 and SKBR3 cells. **B**–**D** Wound healing assays were conducted to investigate the influence of bestatin on cell migration capabilities. **E**–**G** Representative images and statistical analysis of colony formation of breast cancer cells upon bestatin treatment. **H**–**J** Representative images and statistical analysis of cell cycle profiles upon bestatin treatment. Each value represents mean ± S.D from separate experiments

Values of IC50 (mM, mean ± SD) for bestatin in MCF-7(2.412 ± 0.373) and SKBR3 (3.078 ± 0.453) cells were obtained from the dose–response curve (Fig. 1A). Bestatin of 0.25 mM was chosen for in vitro studies as it is a non-toxic concentration. To assess the effects of bestatin on breast cancer cells, wound healing assay was performed. Compared with control group, the open wound of the bestatin treated group was obviously narrowed at 24 h for both MCF-7 and SKBR3 cells. Specifically, the cell migration abilities were inhibited 1.7- and 3.2-fold, respectively (Fig. 1B–D). For the colony formation assay (Fig. 1E–G), the colony diminished after bestatin treatment, indicating the decreased proliferating ability of a single cell to form a cell mass and the breast cancer stem cells with higher proliferating ability might be more sensitive to bestatin. Flow cytometric analyses were performed to confirm whether bestatin can induce cell cycle arrest. We found that bestatin induced cell cycle arrest mainly at G2 phase for MCF-7 cells while arrest G2 phase for SKBR3 cells (Fig. 1H–J). Thus, our results suggest that bestatin can inhibit the migration and proliferation of breast cancer cells.

### Bestatin decreases the stemness of breast cancer stem cells

**Fig. 2:**
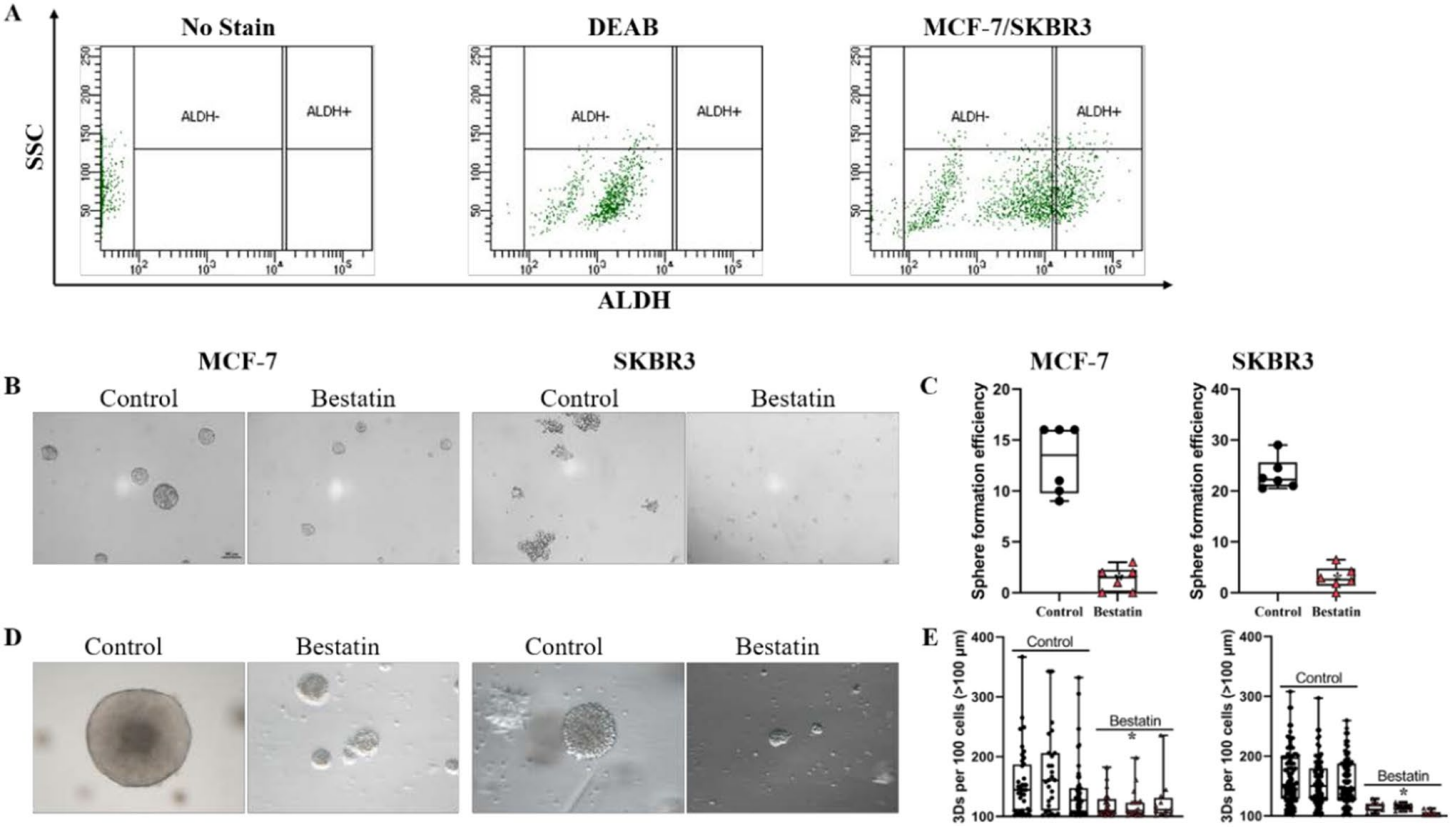
Effects of bestatin on the stemness of breast cancer stem cells. **A** ALDH activities breast cancer cells treated with or without specific ALDH inhibitor diethylaminobenzaldehyde (DEAB) were analyzed by flow cytometry. **B**–**C** Representative images and statistical analysis of the sphere formed by BCSCs sorted from MCF-7 and SKBR3 breast cancer cells. **D**–**E** Representative images and statistical analysis of 3Ds formed by BCSCs sorted from MCF-7 and SKBR3 breast cancer cells. Each value represents mean ± S.D from separate experiments

Putative breast cancer stem cells (BCSCs) were isolated from MCF-7 and SKBR3 cells respectively with the marker of aldehyde dehydrogenase (ALDH) by flow cytometry. Cells treated with ALDH inhibitor 4-(Diethylamino) benzaldehyde (DEAB) was chosen as ALDH^−^ population, and cells sorted from ALDH^+^ population were the highly enriched BCSCs (Fig. 2A). Mammary sphere formation assay was performed to assess self-renewal potential of BCSCs in vitro, and data showed that mammary sphere formation ability was suppressed by bestatin as evident by the decrease of sphere number and size (Fig. 2B, C). When BCSCs isolated from MCF-7 and SKBR3 cells were cultured in the 3D Matrigel for differentiation, bestatin also decreases the differentiation ability of BCSCs for 3D number and size (Fig. 2D, E). Therefore, these results demonstrate that bestatin decreases the stemness of BCSCs.

### Bestatin attenuates the expression of PSA, down-regulates PCNA and increases apoptosis of BCSCs enriched spheres

**Fig. 3:**
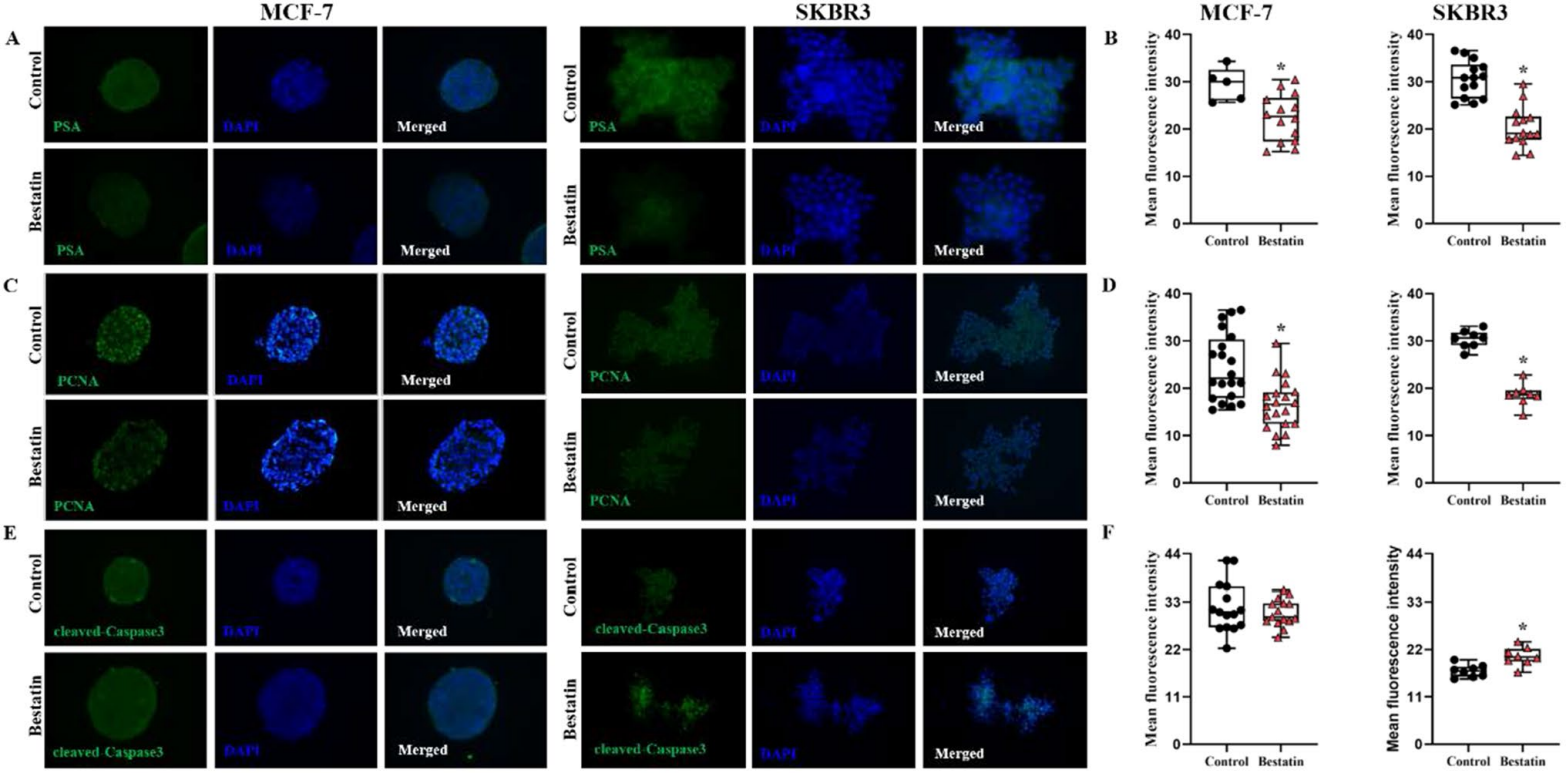
Effects of bestatin on PSA, PCNA and cleaved-Caspase3 expression of spheres formed by breast cancer stem cells. **A**–**B** Immunofluoresence staining and statistical analysis of PSA in control and bestatin-treated BCSC-formed spheres. **C**–**D** Immunofluoresence staining and statistical analysis of PCNA in control and bestatin-treated BCSC-formed spheres. **E**–**F** Immunofluoresence staining and statistical analysis of cleaved-Caspase3 in control and bestatin-treated BCSC-formed spheres. Each value represents mean ± S.D from separate experiments

**Fig. 4:**
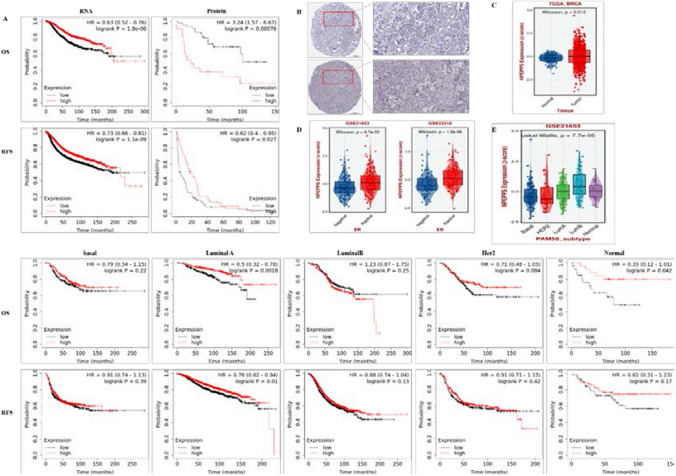
Relationship between PSA expression and breast cancer. **A** Decreased expression of PSA indicated poor prognosis (Overall survival) and RFS (Recurrence free survival) in gene and protein level. **B** The results of Immunohistochemistry between normal and breast cancer tissues. **C** Differential expression of PSA between tumor and normal tissues. **D**–**E** Differential expression levels of PSA gene in different subtypes of breast cancers

As an integral non-transmembrane enzyme, puromycin-sensitive aminopeptidase (encoded by NPEPPS) PSA works to catalyze the cleavage of amino acids of the N-terminus of polypeptides [19]. We further assessed PSA expression level of bestatin on BCSCs enriched spheres. Spheres formed by BCSCs from bestatin -treated group showed lower PSA expression levels for both MCF-7 (22.41 ± 3.50 vs. 29.42 ± 3.69) and SKBR3 (30.55 ± 3.34 vs. 20.40 ± 4.73) (Fig. 3A, B). We also used immunohistochemistry to detect proliferating cell nuclear antigen (PCNA) and cleaved-Caspase3, which are markers of cell proliferation and apoptosis, separately. Bestatin down-regulated PCNA expression in the BCSCs enriched spheres sorted from MCF-7 and SKBR3 cells (Fig. 4C, D), and increased apoptosis by up-regulating active caspase3 expression in the BCSCs enriched spheres sorted from SKBR3 cells (Fig. 3E, F).

### PSA is overexpressed in human breast cancer

For identification of PSA/NPEPPS expression in human breast cancers, KM Plotter database was used for overall survival analysis in gene (RNA-seq) and protein level. It was found that high expression of PSA was associated with better overall survival (OS) rate from Breast RNA-seq data in KM Plotter database (HR = 0.75, Logrank P = 0.015), while with worse OS rate from Breast protein data of Tang_2018 in KM Plotter database (HR = 3.24, Logrank P = 0.00076) (Fig. 4A). Immunohistochemical analysis obtained by the human protein atlas (www.proteinatlas.org) showed that PSA had higher expression levels in tumor tissues compared to normal tissues (Fig. 4B). Consistently, the expression levels of PSA gene from the BEST database was significantly higher in tumor than in normal breast tissues (*p* = 0.012) (Fig. 4C). Furthermore, the expression levels of PSA gene from ER positive breast cancer was significantly higher than ER negative breast cancer analyzed by the BEST database (Fig. 4D). For PSA expression among different prediction analysis of microarray 50 (PAM50) subtype tumors, luminal B breast cancer showed relatively higher expression of PSA (Fig. 4E). The results implied that PSA overexpression might play an important role in the occurrence and development of breast cancer.

### Bestatin inhibits tumorigenesis of BCSCs in vivo

**Fig. 5:**
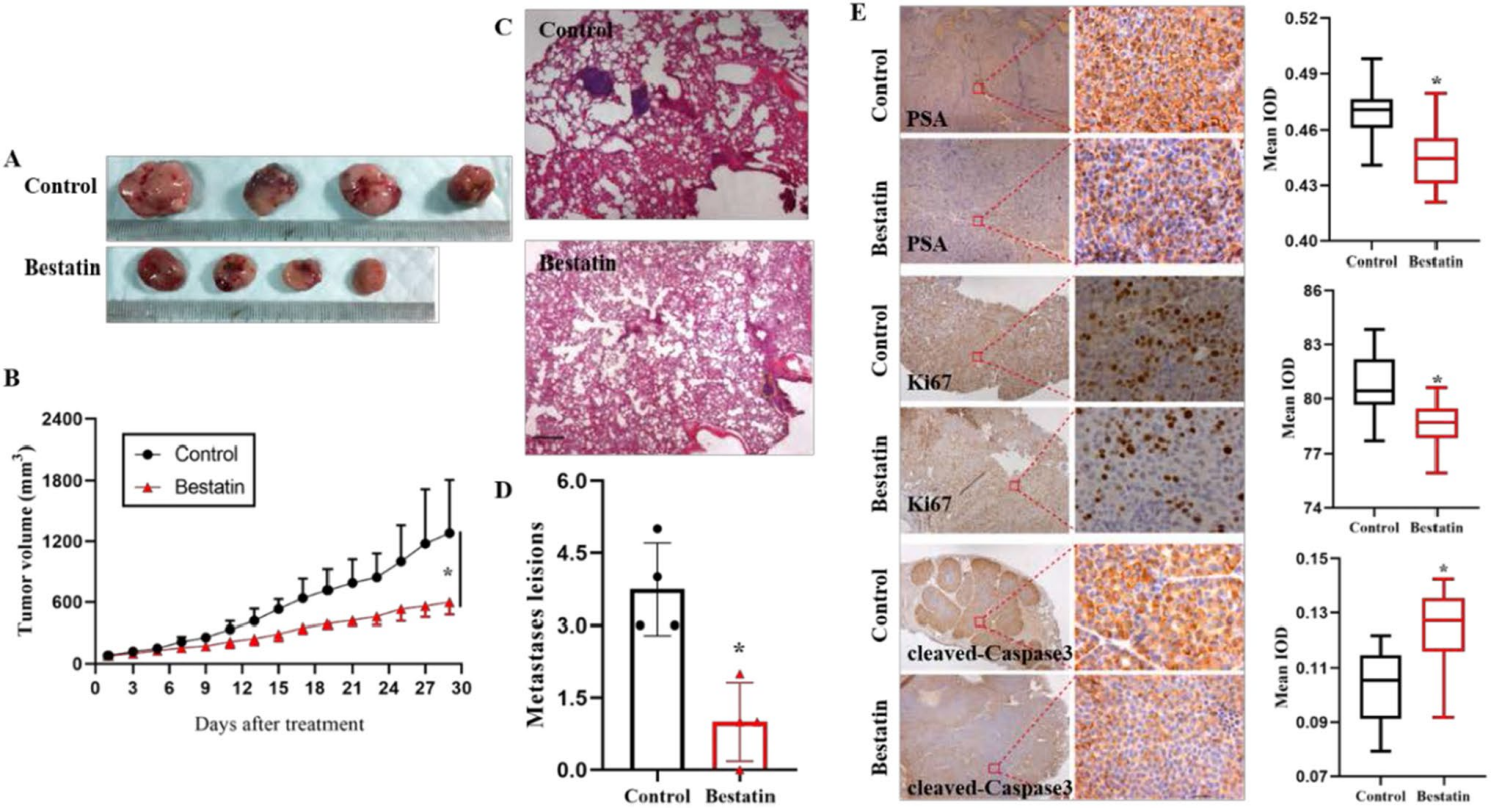
Effects of bestatin on tumorigenesis of BCSCs in vivo. **A**–**B** The images and statistical analysis of tumors derived from BCSCs treated with or without bestatin. **C**–**D** The images and statistical analysis of lung metastasis from the xenografted mice. **E** Immunohistochemistry staining and statistical analysis of PSA, Ki67 and cleaved-Caspase3 in control and bestatin-treated BCSC-formed spheres

A xenograft model was established to determine the role of bestatin in BCSCs. Consistently, bestatin effectively reduced tumor volume in a time-dependent manner, bestatin treated tumor masses grew lower and smaller than the control mice (Fig. 5A, B). Furthermore, bestatin treated tumor masses showed less metastatic lesions in the lungs of mice (Fig. 5C, D). Furthermore, Protein levels of PSA and Ki67 of the xenograft tumor also decreased for bestatin treated group, and cleaved-Caspase3 was up-regulated accordingly (Fig. 5E). Collectively, these results suggested that bestatin may target BCSCs by modulating PSA in vivo.

## Discussion

Bioinformatics analysis, and immunohistochemical staining results revealed that the expression of PSA in breast cancer was significantly higher than that in adjacent tissues. Meanwhile, the expression level of PSA was inversely correlated with OS of breast cancer patients and tumor size of xenografted mice. Therefore, abnormal expression of PSA may play an important role in breast cancer initiation, development and progression.

As a puromycin-sensitive aminopeptidase, PSA/NPEPPS is located in both cytoplasm and cell membranes. PSA is proven to be involved in cell division and differentiation, such as mitosis, meiosis, embryogenesis, reproduction, lipid metabolism and major histocompatibility complex class I peptide processing [19, 25–27]. As a kind of highly conserved protein, PSA has been proven to be associated with some diseases [18–20]. The inhibition of spheres in bestatin-treated breast cancer stem cells reveals that PSA also plays a role in cancer stem cell differentiation. Therefore, inhibiting the PSA expression of cancer stem cells may be a potential choice of cancer therapy.

We revealed that bestatin inhibited tumorigenesis of BCSCs by inhibiting PSA, which might reveal an undiscovered mechanism targeting cancer stem cells. Breast cancer is a kind of heterogeneous tumor with high malignancy, and BCSCs with undifferentiated and self-renewable state are a sub-population of breast cancer cells [28]. BCSCs are characterized by the surface markers such as ALDH, CD133, CD49f, CD44 [29–31]. Higher expression of ALDH activity has been shown to be associated with tumor formation in various types of cancers [32, 33]. Meanwhile, further investigation is needed to confirm the impact of bestatin on inhibiting tumorigenesis in vivo for SKBR3 cells.. Our results revealed that ALDH positive BCSCs are responsible for self-renewal, differentiation and tumor formation. Therefore, ALDH is an effective marker for BCSC isolation.

The dysfunction of cell cycle regulation plays an important role in cell proliferation and results in development of tumor. Programmed cell proliferation is controlled by G1, S, G2 and M phases. Bestatin is found to be able to inhibit tumor growth by inducing cell cycle arrest in colorectal cancer patient [34]. Therefore, the effects of bestatin on the cell cycle of BCSCs were investigated. Our results suggested that bestatin disturbed the cell cycle regulation, which induced increased cell apoptosis subsequently. Further studies are needed to explore the mechanism of PSA regulating BCSC apoptosis.

In conclusion, bestatin inhibits the migration and proliferation of breast cancer cells by decreasing the stemness of BCSCs in vitro and in vivo, and PSA participates in the mechanism through cell cycle regulation, which following induces increased cell apoptosis and decreased cell proliferation of BCSCs. Our study suggests that cancer stem cell can be used as an effective target in breast cancer research, providing new ideas of treatment strategies for breast cancer patients.

## Data Availability

All data and materials are available with the corresponding author.
